# Erectile Dysfunction Preceding Clinically Diagnosed *α*-Synucleinopathies: A Case-Control Study in Olmsted County

**DOI:** 10.1155/2019/6303945

**Published:** 2019-04-09

**Authors:** Shemonti Hasan, Michelle M. Mielke, J. Eric Ahlskog, James Bower, Pierpaolo Turcano, Rodolfo Savica

**Affiliations:** ^1^Department of Neurology, Mayo Clinic, Rochester, MN, USA; ^2^Division of Epidemiology, Department of Health Sciences Research, Mayo Clinic, Rochester, MN, USA

## Abstract

**Objective:**

Autonomic symptoms are common in *α*-synuclein disorders: multiple system atrophy (MSA), Parkinson's disease (PD), dementia with Lewy bodies (DLB), and Parkinson's disease with dementia (PDD). These symptoms may precede the motor findings/clinical diagnosis by years. Erectile dysfunction (ED) is an autonomic symptom that has rarely been studied in these *α*-synuclein disorders. In this population-based, case-control study, we investigated the association between premonitory erectile dysfunction surfacing prior to the clinical-motor manifestations of these *α*-synucleinopathies.

**Methods:**

We used the medical record-linkage system of the Rochester Epidemiology Project to identify cases of *α*-synucleinopathies in Olmsted County from 1991 to 2010. Each male case was matched by age (±1 year) of symptom onset and sex to a control. We reviewed complete medical records of cases and controls to detect erectile dysfunction prior to the clinical-motor onset of *α*-synucleinopathies of any type. We used conditional logistic regression to calculate the odds ratio of all *α*-synucleinopathies, as well as by type, adjusting for diabetes, coffee, and smoking.

**Results:**

A history of male erectile dysfunction was associated with 1.5-fold increased odds of an *α*-synucleinopathy diagnosis of any type in univariate analyses (*p*=0.06). When stratifying *α*-synucleinopathies by type, early erectile dysfunction was most frequent in MSA cases than matched controls (45% vs. 9%). Premotor phase ED was next most frequent among the DLB cases (46% vs. 27% among the controls; OR = 2.83, *p*=0.03; when adjusted for diabetes, smoking, and coffee, OR = 2.98, *p*=0.04). Premotor phase ED was not significantly associated with PD or PDD.

**Conclusions:**

Early erectile dysfunction may be a premotor symptom of MSA and DLB, reflecting premonitory dysautonomia. It was not associated with premotor PD or PDD.

## 1. Introduction

Alpha-synuclein inclusions are the pathological hallmark of a group of neurodegenerative diseases including Parkinson's disease (PD), Parkinson's disease dementia (PDD), dementia with Lewy bodies (DLB), and multiple system atrophy (MSA) [1]. These four disorders have a very prolonged course, and the *α*-synuclein neuropathology typically begins years before the clinical-motor onset [2–4]. However, nonmotor symptoms may surface during the preclinical phase, and this includes autonomic dysfunction [5–8]. In fact, limited dysautonomia may predate the motor symptoms by up to 20 years [5, 9].

Dysautonomia is common among all the *α*-synucleinopathies. Although erectile dysfunction (ED) is an autonomic symptom, it has largely been overlooked among these disorders. Few studies have documented the frequencies of ED in *α*-synucleinopathies: they have been reported as more frequent in PD patients than controls and preceding the PD diagnosis [7, 10]. ED has also been observed in DLB and MSA [7, 11].

The focus of this study is on the *α*-synucleinopathy premotor phase, which typically precedes the clinical diagnosis by years. To what extent is ED documented during this premotor phase in these four *α*-synucleinopathies? Obviously, early diagnosis may be facilitated by recognition of such early autonomic symptoms. Herein, we assess the frequency of early (premotor) ED in these four *α*-synuclein neurodegenerative syndromes, using a population-based, case-control design [12, 13].

## 2. Methods

### 2.1. Cases

We used the medical-record linkage system Rochester Epidemiology Project (REP) to identify residents of Olmsted County, Minnesota, who were clinically diagnosed with parkinsonism-associated *α*-synucleinopathies between 1991 and 2010. Details on case identification are reported elsewhere [14–16]. The REP indexes all medical information of individuals residing and receiving care in Olmsted County, Minnesota [17–20]. All medical diagnoses, surgical interventions, and procedures are indexed into the computerized system using the Hospital Adaptation of the International Classification of Diseases, Eight Revision (H-ICDA), and the International Classification of Diseases, Ninth Revision (ICD-9) [21, 22]. Our diagnostic criteria for parkinsonism included two steps: defining parkinsonism as a syndrome and defining the type of parkinsonism within that syndrome. We defined parkinsonism as having at least two of the four following cardinal signs: rest tremor, bradykinesia, impaired postural reflexes, and cogwheel rigidity.

We defined PD as parkinsonism that satisfied the three following criteria: (1) no other cause (e.g., repeated stroke with stepwise progression, repeated head injury, history of encephalitis, neuroleptic treatment within 6 months before onset, hydrocephalus, and brain tumor); (2) no documented unresponsiveness to levodopa at doses of at least 1 g/day in combination with carbidopa (applied only to treated patients); (3) no prominent or early signs of extensive nervous system involvement (e.g., dysautonomia) not explained otherwise [23]. Previously published consensus criteria were used to diagnose MSA, DLB, and PDD [14, 16, 24, 25]. All of the cases were reviewed, and the clinical diagnoses were finalized by a movement disorders specialist (RS). The index date was defined as the year in which a cardinal sign was first described by the patient, family member, or care provider in the medical record. Our case-finding procedure has proven to be reliable [14, 15]. Cases of *α*-synucleinopathies included patients with PD, DLB, PDD, and MSA.

### 2.2. Controls

Cases were matched by sex and age (±1 year) to a referent living in Olmsted County, Minnesota, who was free of parkinsonism prior to the index date (the year of onset of the parkinsonism). Matched controls with other neurologic diseases were not excluded from the study. The REP provided a list of all county residents from whom matched controls were randomly drawn. A nurse abstractor reviewed the potential controls and marked any who had a possible diagnosis of parkinsonism. These marked controls were reviewed by a neurologist (RS) to exclude the diagnosis of any type of parkinsonism before the index date in the matched case. Any cases that had a diagnosis on or before the index date were included as a case and replaced with a randomly drawn referent individual from the general population.

### 2.3. Ascertainment of Erectile Dysfunction

ED was ascertained up to the time of the initial *α*-synucleinopathy motor sign(s) for the cases and up to the index date of the controls. ED analysis did not go beyond the index date. ED was limited to males and defined as having a diagnosis of “erectile dysfunction” or “impotence” in the medical record or having the code “impotence” or “erectile dysfunction” (ICD-9 code 607.84). If there was such a code, we reviewed the medical record for the date of the specific code to confirm the presence of a diagnosis of ED. Transient ED that the clinician attributed to exposures to medications was not counted. Complete medical records were used to abstract the exposure, which was performed by a medical student (SH) who was blinded to the case/control assignment. The information was abstracted from the first medical record available to the index date. The date of initial ED diagnosis per the patient or per the medical record if the patient did not know was recorded as the date of ED onset.

### 2.4. Data Analysis

We used conditional logistic regression to calculate the odds ratio (OR) of having ED (males only) among the cases compared to the controls, a 95% confidence interval, and a *p* value (two-tailed test, *α* = 0.05). For the second model, we adjusted for diagnosis of types 1 or 2 diabetes mellitus as a possible confounder for ED, and for smoking (ever versus never) and coffee (ever versus never) as these have been shown to be associated with Parkinson's disease [26–29]. These adjustments were done using stepwise selection. We then stratified each variable by the type of *α*-synucleinopathy. We also calculated the median time from ED diagnosis to the onset of motor symptoms for each *α*-synucleinopathy. All statistical analyses were performed using JMP version 13.0.0 and R version 3.4.2.

### 2.5. Standard Protocol Approvals, Registration, and Patient Consent

The study was approved by the institutional review boards at Mayo Clinic and Olmsted Medical Center. Written informed consent was not required for passive medical record review.

## 3. Results

We identified 280 male patients who developed parkinsonism due to a clinically diagnosed *α*-synucleinopathy between 1991 and 2010. They were matched to 280 male control subjects by age (±1 year). Among these 280 case-control pairs, the median age at index date was 75 years (IQR: 13.0).

Among the 280 male cases, 185 (66.1%) had a diagnosis of PD; 56 (20.0%) had DLB; 27 (9.64%) had PDD; 12 (4.29%) had MSA (Supplementary Table 1). ED had been diagnosed in 92 cases (32.5%) and 70 controls (24.7%), yielding an OR of 1.47 (95% CI: 0.99–2.16, *p*=0.06) among all *α*-synucleinopathies (Table 1). This finding remained insignificant after adjusting for diabetes, smoking, and coffee (*p*=0.06).

When stratifying by type of *α*-synucleinopathy, the MSA cases had the highest percentage of ED (45%; 5/12). Conditional logistic regression analysis could not be done, given only one control subject with ED (9%; 1/12); however, this was significant using McNemar's test, *X* ^2^ = 4.5, *p*=0.03.

The next highest percentage of ED cases was identified in the DLB group (46.4%; 26/56). In the DLB case-control univariate analysis, the difference was significant (OR: 2.83; 95% CI: 1.12–7.12; *p*=0.03). The *p* value remained significant (*p*=0.04) after adjusting for diabetes, smoking, and coffee in the conditional logistic regression analysis.

The percentage of ED in PD (27.8%; 52/185) and PDD (33.3%; 9/27) individuals were substantially less than in the MSA and DLB groups. The case-control analysis revealed no significant differences in these two *α*-synucleinopathy disorders (Table 1).

**Figure 1:**
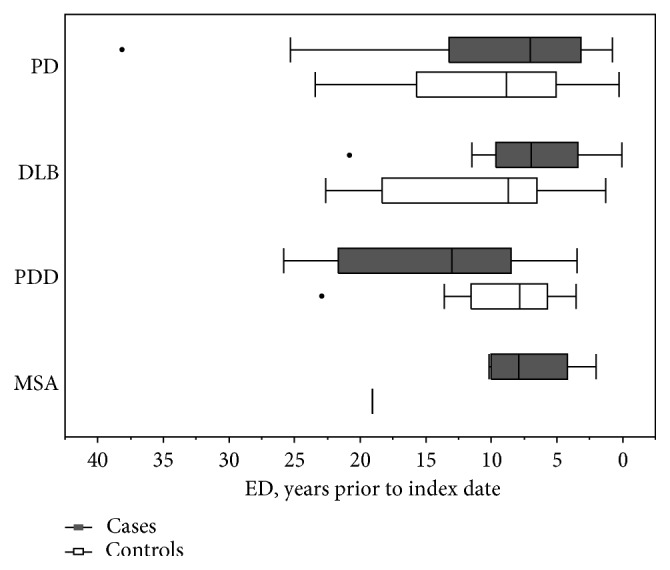
Distribution of lag times between onset of ED and index date for *α*-synucleinopathies.

**Figure 2:**
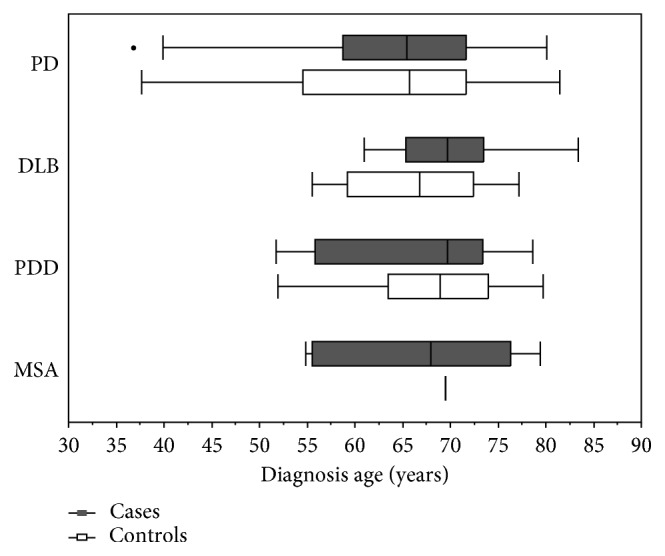
Distribution of age of ED onset for *α*-synucleinopathies.

The median time from ED diagnosis to the onset of any *α*-synucleinopathy was 7.73 years (IQR: 8.30) among the cases, compared to 8.57 years prior to the index year (for controls (IQR: 10.46; Wilcoxon rank-sum test, *p*=0.17). The PD, DLB, and MSA cases had slightly shorter distribution of lag times compared to controls, but not for PDD (Figure 1). However, these differences were not significant. For those with ED, the median age of ED onset for any *α*-synucleinopathy was 66.41 years (IQR: 10.32) and 66.47 years for the controls (IQR: 12.59; Wilcoxon rank-sum test, *p*=0.36). The median age of ED onset between each *α*-synucleinopathy and their matched controls was not statistically significant (Figure 2).

## 4. Discussion

The premotor phases of Parkinson's disease (PD) and Parkinson's disease dementia (PDD) have been clinically [5, 6, 30] and neuropathologically [2–4] characterized, typically spanning many years; dysautonomic symptoms (especially constipation [5]) may surface during this prolonged premotor phase. The premotor phases of the other two *α*-synucleinopathies (MSA and DLB) have been less well characterized, but presumably also spanning many months to many years. Given that clinical and pathological evidence of dysautonomia has been a hallmark of the PD and PDD premotor phase, we assessed whether another autonomic symptom, ED, surfaced before the clinical picture of these *α*-synuclein disorders became apparent.

Our findings revealed that ED is a frequent early (premotor) symptom among not only MSA cases (41.7%) but also DLB cases (46.4%). However, it was not significantly found among PD or PDD cases during the premotor phase. It may seem somewhat counterintuitive that DLB, but not PD or PDD, was associated with early ED. However, this finding is consistent with a prior report documenting significantly more prominent dysautonomia in DLB than PD, but not as severe as in MSA [31].

Although we did not find an association between ED and odds of developing PD or PDD, the Health Professionals Follow-Up Study did report an association between early ED and PD [10]. This was a retrospective questionnaire study. Whether it was affected by recall bias can be debated. In addition, the diagnosis of PD was based on the date of diagnosis rather than the date of motor-symptom onset as seen in our study. A separate issue relates to the prevalence of ED in each of these *α*-synuclein disorders during the established disease phase. We did not assess ED beyond the index date.

Our study has a number of strengths. First, this study included all incident cases of *α*-synucleinopathies within a defined population; thus, the risk of selection bias is minimized. Second, this study used incident cases of *α*-synucleinopathy by marking the index date as the time of symptom onset. By reviewing the medical records, we retrodated the exposure; thus, incidence-prevalence bias is reduced. Third, this study population also used randomly drawn residents without a confirmed *α*-synucleinopathy from the general population of Olmsted County, thus reducing referral bias. Fourth, our study used a medical-record linkage system to access historical records that have patient information for several decades, allowing us to abstract exposures that occurred early in life. Lastly, our study examined the association of the different types of clinically diagnosed *α*-synucleinopathies with ED. We also adjusted for other potential factors associated with ED, such as diabetes mellitus, and with *α*-synucleinopathies, such as coffee consumption and smoking [26–29, 32, 33].

However, our study also has limitations. First, the sample sizes for each type of *α*-synucleinopathy were small, particularly for MSA. Second, ED, especially mild ED, is likely underreported. Physicians are unlikely to inquire about it, and men may be reluctant to report it. The ED documented in our medical records presumably reflected more problematic ED. Regardless, ED underreporting should have been equally distributed among both case and control groups; recall bias should not be present with this medical-record study design. Third, the diagnoses of the cases were based on clinical, not pathological findings. However, the clinical-pathological discrepancies within our cohort of individuals who have developed parkinsonism in Olmsted County from 1991 to 2010 have been studied. Of the patients with neuropathologic examination at autopsy (*n*=60, 9%), clinical-pathologic concordance was found in 52 (86.7%) of the cases and discrepancies were found in 8 (13.3%) [34]. Lastly, this study did not include female sexual dysfunction, therefore not evaluating premotor dysautonomia that may occur in women.

To summarize, early ED among patients destined to develop MSA or DLB presumably reflects early *α*-synuclein involvement of the autonomic nervous system. For these disorders, ED may be a premonitory diagnostic clue to the diagnosis along with other dysautonomia symptoms as well as REM sleep behavior.

## Figures and Tables

**Table 1:** Association between *α*-synucleinopathies and preceding erectile dysfunction.

Type	Cases	Controls	Primary model		Secondary model	
			OR (95% CI)	*p* value	OR (95% CI)	*p* value
All *α*-synucleinopathies	92 (32.6%)	70 (25.0%)	1.47 (0.99–2.16)	0.06	1.49 (0.99–2.24)	0.06
PD	52 (27.8%)	44 (23.8%)	1.19 (0.74–1.90)	0.47	1.31 (0.78–2.18)	0.31
DLB	26 (46.4%)	15 (26.8%)	2.83 (1.12–7.19)	0.03	2.74 (1.04–7.21)	0.04
PDD	9 (33.3%)	10 (37.0%)	0.8 (0.22–2.98)	0.74	0.422 (0.07–2.54)	0.34
MSA	5 (41.7%)	1 (8.3%)	—	0.03^*∗*^	—	—

Primary model: unadjusted. Secondary model: adjusted for diabetes, smoking (ever versus never), and coffee (ever versus never) using forward stepwise selection. ^*∗*^Because there were not enough individuals in the MSA control group with ED, we could not do conditional logistic regression. Therefore, we used McNemar's test to calculate if MSA cases were different than the controls. MSA *X* ^2^ = 4.5.

## Data Availability

The demographic data used to support the findings of this study are included within the article.

## Supplementary Materials

Supplementary Materials Supplementary Table 1: Demographics of Cases.
