# Supplementary material for: Erectile Dysfunction Preceding Clinically Diagnosed α-Synucleinopathies: A Case-Control Study in Olmsted County
Source: Parkinsons Dis. 2019 Apr 9;2019:6303945. doi: 10.1155/2019/6303945 (PMC6481034; doi:10.1155/2019/6303945)
Supplement: Supplementary Materials — Supplementary Table 1: Demographics of Cases. [file 6303945.f1.pdf]

*

Supplementary Table 1-Demographics of Cases

**Type of α-synucleinopathy**	**Frequency (%)**
Parkinson's disease	185 (66.1%)
Lewy Body Dementia	56 (20.0%)
Parkinson's Disease-Dementia	27 (9.64%)
Multiple System Atrophy	12 (4.26%)
**Race**	
White	272 (96.5%)
Asian	7 (2.48%)
Black	2 (0.71%)
Other/Mixed	1 (0.36%)
